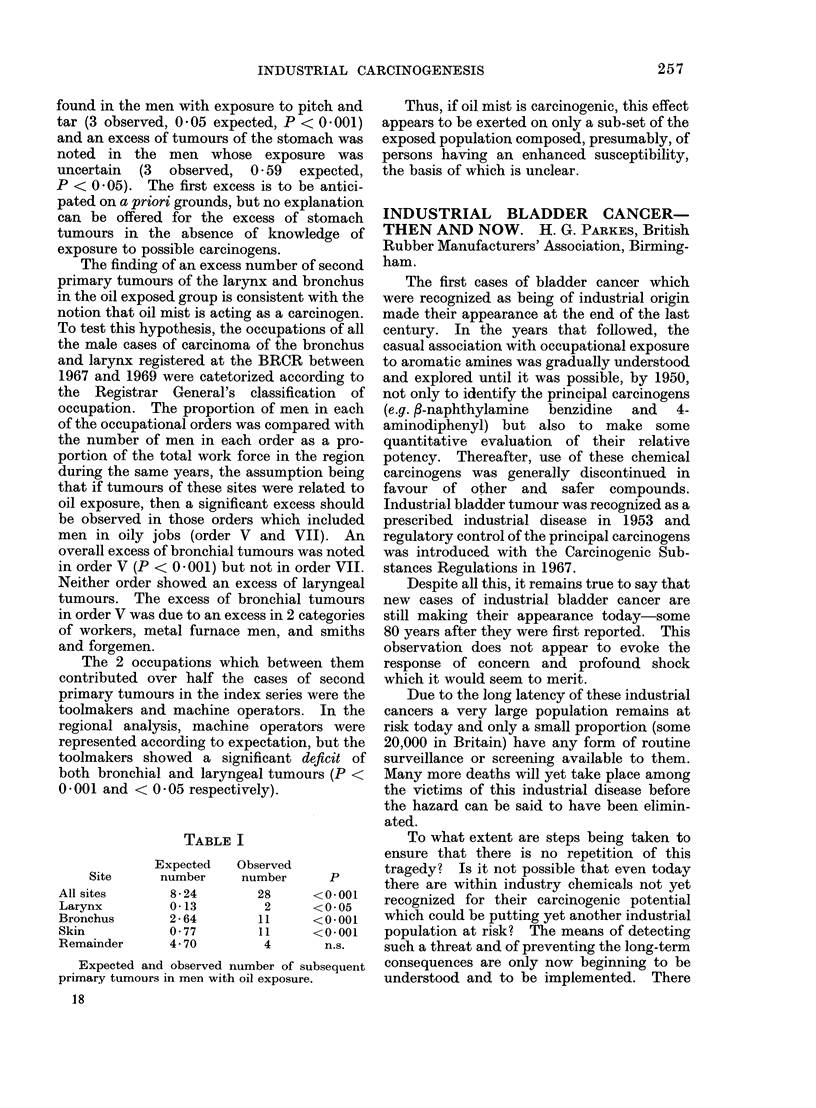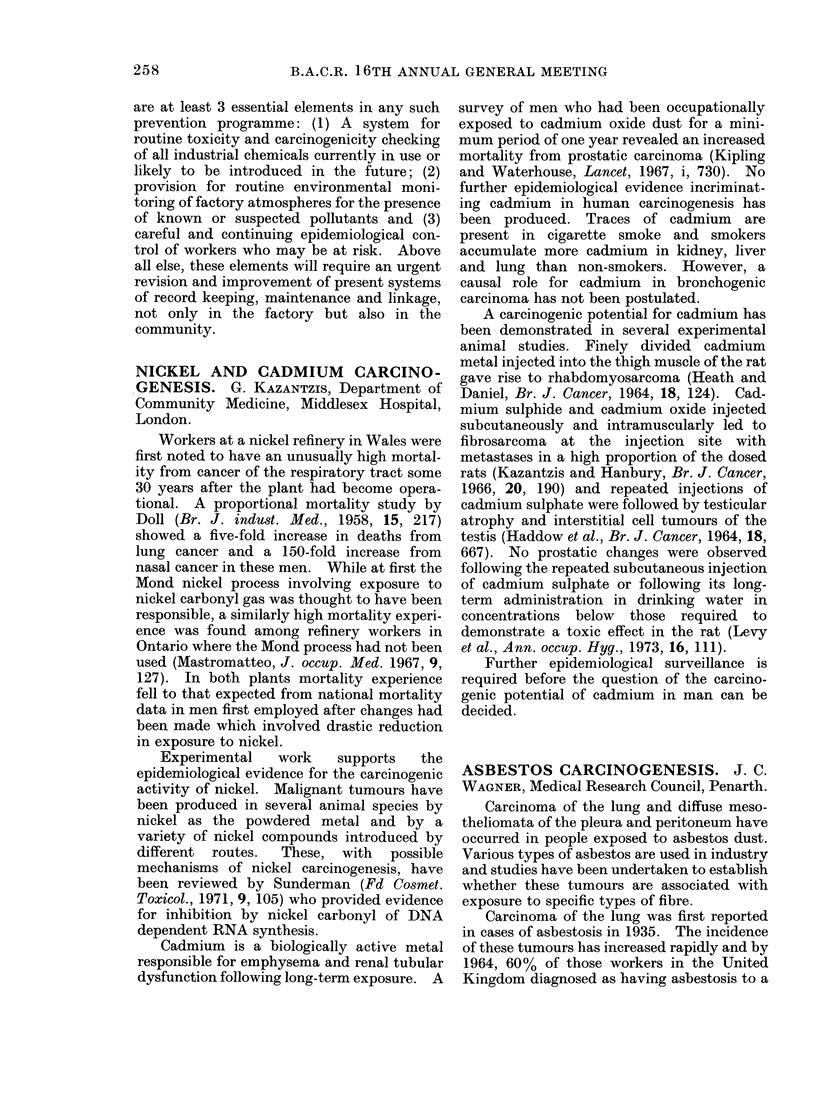# Proceedings: Industrial bladder cancer--then and now.

**DOI:** 10.1038/bjc.1975.204

**Published:** 1975-08

**Authors:** H. G. Parkes


### INDUSTRIAL BLADDER CANCER—THEN AND NOW.

H. G. PARKES, British Rubber Manufacturers' Association, Birmingham.

The first cases of bladder cancer which were recognized as being of industrial origin made their appearance at the end of the last century. In the years that followed, the casual association with occupational exposure to aromatic amines was gradually understood and explored until it was possible, by 1950, not only to identify the principal carcinogens (*e.g.* β-naphthylamine benzidine and 4-aminodiphenyl) but also to make some quantitative evaluation of their relative potency. Thereafter, use of these chemical carcinogens was generally discontinued in favour of other and safer compounds. Industrial bladder tumour was recognized as a prescribed industrial disease in 1953 and regulatory control of the principal carcinogens was introduced with the Carcinogenic Substances Regulations in 1967.

Despite all this, it remains true to say that new cases of industrial bladder cancer are still making their appearance today—some 80 years after they were first reported. This observation does not appear to evoke the response of concern and profound shock which it would seem to merit.

Due to the long latency of these industrial cancers a very large population remains at risk today and only a small proportion (some 20,000 in Britain) have any form of routine surveillance or screening available to them. Many more deaths will yet take place among the victims of this industrial disease before the hazard can be said to have been eliminated.

To what extent are steps being taken to ensure that there is no repetition of this tragedy? Is it not possible that even today there are within industry chemicals not yet recognized for their carcinogenic potential which could be putting yet another industrial population at risk? The means of detecting such a threat and of preventing the long-term consequences are only now beginning to be understood and to be implemented. There are at least 3 essential elements in any such prevention programme: (1) A system for routine toxicity and carcinogenicity checking of all industrial chemicals currently in use or likely to be introduced in the future; (2) provision for routine environmental monitoring of factory atmospheres for the presence of known or suspected pollutants and (3) careful and continuing epidemiological control of workers who may be at risk. Above all else, these elements will require an urgent revision and improvement of present systems of record keeping, maintenance and linkage, not only in the factory but also in the community.